# Simulation on the Evolution Trend of the Urban Sprawl Spatial Pattern in the Upper Reaches of the Yangtze River, China

**DOI:** 10.3390/ijerph19159190

**Published:** 2022-07-27

**Authors:** Yuxiang Zhang, Dongjie Guan, Xiujuan He, Boling Yin

**Affiliations:** 1 School of Smart City, Chongqing Jiaotong University, No. 66 Xuefu Road, Nan’an District, Chongqing 400074, China; 622200023003@mails.cqjtu.edu.cn (Y.Z.); xjhe722@163.com (X.H.); 18323254998@163.com (B.Y.); 2 State Key Laboratory of Mountain Bridge and Tunnel Engineering, Chongqing Jiaotong University, No. 66 Xuefu Road, Nan’an District, Chongqing 400074, China

**Keywords:** urban sprawl, the upper Yangtze River, cold–hot-spot pattern, USSA model, scenario simulations

## Abstract

Urban sprawl has become the main pattern of spatial expansion in many large cities in China, and its ecological and environmental effects profoundly impact Chinese urban development. In this paper, nighttime light data and statistical yearbook data are adopted as basic data sources to simulate the evolution trend of the urban sprawl in the upper Yangtze River (UYR), China. First, the urban sprawl index (USI) is employed to assess the level of urban sprawl and to determine the characteristics of urban sprawl under different scales. Second, the spatial autocorrelation model is applied to reveal the spatial pattern change characteristics of urban sprawl from 1992 to 2015. Third, a scenario analysis model of urban sprawl is constructed to simulate the evolution trend of the urban sprawl under different scenarios. Finally, based on the Geodetector, the influence of factors and factor interactions influencing urban sprawl in different time periods is analyzed. The results yield the following main conclusions: (1) The urban sprawl in the UYR first intensifies and then stabilizes over time. The number of cities with high USI in Sichuan province, medium cities, and Chengdu-Chongqing urban agglomeration increases over time, indicating that urban sprawl is intensifying in these areas. (2) The urban sprawl hot spots experience a pattern transformation process of point-like expansion-point-ring expansion-point-axis expansion-axis radiation. (3) Under the scenarios with different scales, the urban land sprawl in large cities is the highest, accounting for more than 47% of the UYR. Urban land sprawl extent in the Chengdu-Chongqing urban agglomeration is the highest, accounting for more than 51% of the UYR. The cities exhibiting the highest sprawl are Chongqing, Lijiang, and Kunming, accounting for 25.84%, 7.37%, and 5.11%, respectively, of the UYR. (4) In the different time scenario simulations, the urban land in large cities exhibits the highest sprawl, accounting for approximately 48.16% of the UYR. The urban land in the Chengdu-Chongqing urban agglomeration demonstrates the highest sprawl, accounting for 50.92% of the UYR. (5) From 1996 to 2002, the driver with the highest influence on urban sprawl was secondary industry share of GDP, with a *q*-statistic of 0.616. From 2009 to 2015, the driver with the highest influence on urban sprawl was green space per capita with a *q*-statistic of 0.396.

## 1. Introduction

Urban sprawl was a noteworthy appearance and problem in the process of urban development in Western countries in the 20th century. At present, there is only a vague definition of the connotation of urban sprawl, but all definitions exhibit one common feature: low density, scattered development, poor accessibility, and a single function [1]. Against the current background of large-scale urbanization, the development of many cities has exceeded the normal track, and the layout of urban construction is seemingly disorderly or even out of control. The phenomenon of urban sprawl has also occurred in China, which is chiefly embodied in the disorderly expansion of built-up land, and the speed of population development is lower than that of land development, which reduces the urban land-use efficiency and triggers impacts on social and economic development, resources, ecology, and the environment [2,3,4]. The contradictions and conflicts between these aspects have seriously harmed the social and economic development of China. Urban sprawl has become the main method of spatial expansion in many large cities in China. Many problems in the development of Western cities have also been manifested to varying degrees in China, which has attracted the attention of relevant scholars and departments [5,6].

Urban sprawl first appeared in the urbanization process of Western developed countries. After World War II, the popularity of motor vehicles and the massive construction of roads led to the expansion of urban space, which destroyed the agricultural land around the cities and the ecological environment inside the cities [7]. The phenomenon of suburbanization dominated by domestic automobiles exacerbated the employment problem and the proliferation of low-density housing, resulting in the problem of urban sprawl. Urban sprawl studies originated in the 1960s. Whyte [8] first proposed the term “urban sprawl” in 1958. The process of suburbanization emerged in France in the 1960s and has since sprawled to the suburbs regardless of the size of the city, with the rate of sprawl varying from region to region [9]. In the 1990s, as the economy grew, a large number of residents chose to migrate to large cities, which exceeded the local housing demand. At this time, a spatial pattern of diffuse cities emerged [10]. At the beginning of the 21st century, governments at all levels greatly accelerated urban sprawl by investing heavily in transportation and other public facilities [11]. Market failures can also exacerbate urban sprawl by affecting population growth, rising incomes, and falling commuting costs [12].

The current research on urban sprawl has mainly concentrated on four aspects. The first aspect is the delimitation of urban sprawl. Whyte [8] delimited urban sprawl as the rapid development of urban suburbs. Gottmann [13] proposed that sprawl is the continuous expansion along the periphery of a metropolis. Anderson et al. [14] understood urban sprawl as the separation of residential land from other land uses because of its location on the urban periphery; a general decrease in land-use intensity; a highly connected transportation network; and the expansion of urban boundaries. It is evident that there exists no very clear definition of the connotation of urban sprawl, but it is found that the definition has changed from neutral to derogatory, and all definitions exhibit a common feature: low density, inaccessibility, scattered development, and functional inefficiency. The second aspect is the measurement study of urban sprawl. Two types of measurements are typically used to measure urban sprawl. One uses a single index to measure a particular aspect of sprawl, and the other uses a composite index to measure multidimensional aspects of sprawl. The most well-known single index is the sprawl index (SI) [15], which was used to measure the sprawl score status of metropolitan areas in the USA in 2000. Ewing et al. [16] originally used a single sprawl index to estimate sprawl in 448 metropolitan counties. The representative of composite index is that Ewing et al. [17] used principal component analysis to screen out 22 highly correlated variables, and on that basis, he formed four factors of residential density, land-use mix, intensity of economic centers and downtown centers, and accessibility of neighborhood networks, which were combined to obtain the sprawl index. Torrens [18] measured urban sprawl in terms of seven aspects, including urban growth, with a total of 42 factors. The composite index method also includes dynamic model, spatial model, measurement model, statistical model, and integrated model. Zhou et al. [19] explored the factors influencing vertical urban sprawl by constructing regression models from the perspective of government, developers, and residents. Das and Angadi [20] combined spatial landscape metrics and the Shannon entropy model to analyze the spatial assessment of urban sprawl. The third aspect is the study of factors influencing urban sprawl mainly from two aspects: natural conditions and socio-economic conditions. Wang et al. [21] analyzed urban decentralization and urban renewal as socioeconomic factors behind urban sprawl in China. Marais et al. [22] argued that historical path dependencies and interdependencies are considered the main factors of urban sprawl. Fan and Zhou [23] believed the factors that positively influence urban sprawl are fiscal competition among governments, competition for investment, and competition to promote the environment. Guite [24] identified population growth and the work sector as contributing to sprawl. Domingo et al. [25] used multiple scenario simulations to reveal the potential impact of planning on urban growth. Due to the emergence of residential density hot spots, Koprowska et al. [26] used hot spot analysis to demonstrate a link between urban sprawl and increased availability of urban green zones. Overall, the impact factors of urban sprawl include natural conditions, government, history, population, economy, etc. The fourth aspect involves the study methods of urban sprawl control. On the basis of the least cumulative resistance model, Guan et al. [27] simulated the urban sprawl conditions under different scenarios by considering the level of the source, ecological barriers, ecological resistance, and urban sprawl and proposed suggestions to guide the sustainable development of cities. Li et al. [28] proposed that the sprawl of megacities in China requires multiple-scale aspects to join together to manage by examining urban regional expansion patterns. Gavrilidis et al. [29] used hierarchical analysis to evaluate green space proposals in urban areas and proposed a methodological framework for controlling urban sprawl. Menzori et al. [30] argued that management capacity affects the geographic location of urban growth and, in turn, the moderation of urban sprawl. Tan et al. [31] concluded that innovation policies are not driving urban sprawl.

The research on urban sprawl is quite comprehensive, covering various aspects such as definition, quantification, mechanism, influence, and regulation. However, the research on urban sprawl has begun to solidify, but from the perspective of the research content, there remains a lack of research in the below two dimensions. The first dimension is the scale effect of urban sprawl, and the second dimension is the simulation and prediction research on urban sprawl. Against the background of Chinese large-scale urbanization, the phenomenon of urban sprawl will occur for a long time. By simulating urban sprawl scenarios, guiding the sustainable development of cities in the above basin is of great importance to the ecological security of the area.

At present, the main methods adopted to simulate urban sprawl include the cellular automata model, neural network algorithm, SLEUTH model, CLUE-S model, and FLUS model. Yang et al. [32] used CA–Markov model to simulate future urban sprawl. Okwuashi and Ndehedehe [33] combined machine learning with cellular automation to build models for simulating future city states. Liao et al. [34] proposed an urban cellular automaton model for predicting urban growth boundaries under different levels of ecological space of importance. Li et al. [35] employed the SLEUTH model to discuss the temporal and spatial variation of urban growth. Huang et al. [36] used CLUE-S model to simulate the urban growth boundary. Chen et al. [37] obtained the characteristics of future urban sprawl based on FLUS model simulations. Some scholars have also used neural network models to identify and predict future urban sprawl scenarios, and the results have achieved a high degree of accuracy [38,39,40,41]. There are also combinations of neural networks and other models to predict urban sprawl [42,43] These models mainly consider social, economic, and policy factors impacting urban sprawl. However, little consideration is given to ecological factors, and the simulation results often lack scale-effect research.

This paper chooses the upper Yangtze River as the research area. The extent of urban sprawl in Sichuan, Chongqing, Yunnan, and Guizhou over the past 20 years and the process of changing patterns of urban sprawl characteristics on multiple scales are measured. The pattern and evolution of urban sprawl cold and hot spots are analyzed, an urban sprawl scenario analysis model is developed on this basis to simulate the evolution trend under different scenarios, and a basis is provided for sustainable city development. Finally, based on the Geodetector, we analyzed the factors influencing urban sprawl and the interaction of factors in the two time periods.

## 2. Study Area and Data Sources

### 2.1. Study Area

**Figure 1:**
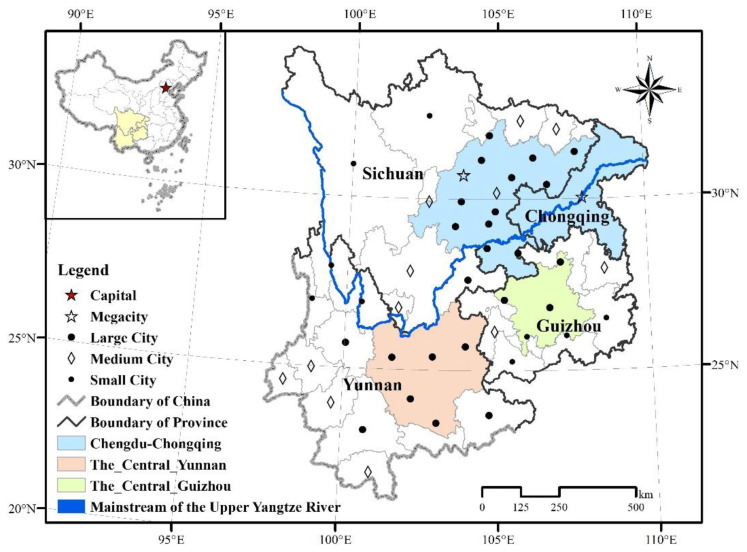
Location of the Upper Yangtze River, China.

The upper Yangtze River (UYR) extends from the source of the Yangtze River to the main stream of Yichang, spanning from 90.54–111.46° E longitude and 24.46–35.75° N latitude, and has a length of 4511 km, accounting for roughly 70% of the entire length of the Yangtze River. The main tributaries are Yalong River, Min River, Jialing River, Wu River, and so on. The terrain is high in the west and low in the east, with the Tibetan Plateau in the west at an altitude of over 6000 m and the plains and hills of the Sichuan Basin in the east at an altitude of only a few hundred meters. The river runoff in the UYR accounts for 48% of the runoff in the entire basin, which determines the changes in the water environment of the entire Yangtze River. Therefore, the ecological protection of the UYR is correlated with the ecological security of the entire basin and the long-term stability and sustainable development of China. This paper mainly studies the four provinces (cities) of Sichuan, Chongqing, Yunnan, and Guizhou in the UYR. There are 47 cities in the study area, including 2 megacities with urban populations over 5 million, 24 large cities with urban populations over 1 million, 12 medium-sized cities with urban populations over 500,000, and 9 small cities with urban populations less than 500,000, as shown in Figure 1. As of the end of 2019, the year-end population of these four provinces (cities) reached 199.8057 million, accounting for 14.27% of the Chinese population, and the regional GDP reached CNY 110,214.466 billion, accounting for 11.12% of the GDP of China.

As shown in Figure 1, the Chengdu-Chongqing urban agglomeration consists of Chongqing and Sichuan provinces (except Beichuan County, Pingwu County, Wanyuan City, Tianquan County, and Baoxing County), with a total area of 185,000 km^2^. The Central Yunnan urban agglomeration is located in the central part of Yunnan Province, consisting of 4 cities in Kunming, Qujing, Yuxi, and Chuxiong and 7 counties and cities in the northern part of Honghe Prefecture, with a total area of 111,400 km^2^, accounting for 29% of the total area of Yunnan Province. The Qianzhong urban agglomeration is located in the central region of Guizhou Province, including the 6 cities (states) of Guiyang, Zunyi, Bijie, Anshun, Qiandongnan, and Qiannan and 33 counties (cities and districts) of Guian New District, with a total area of 53,800 km^2^. The three urban agglomerations, as the 19 urban agglomerations fostered by the government, are still subject to systematic ecological risks with the growth of urban sprawl intensity and the serious threat of landscape ecosystem degradation.

### 2.2. Data Sources

The adopted DMSP/OLS nighttime light data are released by the National Physical Earth Data Center (http://www.geodata.cn/). This type of data is monitored by six satellites equipped with DMSP/OLS sensors. The longitude range covered by the data is from −180° to 180°, the latitude range is from −65° to 75°, and the spatial resolution is 30 arc seconds. These data remove the influences of sunlight, aurora, etc., and they more accurately reflect the nighttime light information, which is beneficial for the quick extraction of urban construction land.

The GIS data adopted in this paper include DEM, NDVI, land cover, farmland production potential, and soil erosion data for the UYR. These data were obtained from the Resource and Environmental Science and Data Center of the Chinese Academy of Sciences (https://www.resdc.cn/). The spatial resolution is 1 km × 1 km. The main railroad data are obtained from OpenStreetMap (https://master.apis.dev.openstreetmap.org/).

The statistical yearbook data contain the urban population of various cities in the UYR from 1992 to 2015, including socioeconomic data. These data originate from the EPS data platform, China Demographic Yearbook, Sichuan Province Statistical Yearbook, Chongqing City Statistical Yearbook, Guizhou Province Statistical Yearbook, and Yunnan Province Statistical Yearbook.

## 3. Methods

### 3.1. Urban Sprawl Index

In this paper, the USI is cited for quantifying the extent of urban sprawl [23,44]. This index takes into account two main indicators that are relevant to urban sprawl: urban land and urban population. The urban sprawl index is calculated as follows:

$${\mathrm{USI}}_{\left(t_{1},t_{2}\right)}^{i}=UA_{\left(t_{1},t_{2}\right)}^{i}-UP_{\left(t_{1},t_{2}\right)}^{i}$$

where ${\mathrm{USI}}_{{(t}_{1}{,t}_{2})}^{i}$ is the USI of city *i* from time *t*_1_ to time *t*_2_; ${\mathrm{UA}}_{{(t}_{1}{,t}_{2})}^{i}$ is the annual growth speed of urban land in the city *i* between *t*_1_ and *t*_2_; ${\mathrm{UP}}_{{(t}_{1}{,t}_{2})}^{i}$ is the annual growth speed of urban population in the city *i* between *t*_1_ and *t*_2_. The formula is calculated as follows:

$$UA_{\left(t_{1},t_{2}\right)}^{i}=\left({\left(\frac{A_{t_{2}}^{i}}{A_{t_{1}}^{i}}\right)}^{\frac{1}{t_{2}-t_{1}}}-1\right)\times 100\%$$

$$UP_{\left(t_{1},t_{2}\right)}^{i}=\left({\left(\frac{P_{t_{2}}^{i}}{P_{t_{1}}^{i}}\right)}^{\frac{1}{t_{2}-t_{1}}}-1\right)\times 100\%$$

where $A_{t_{1}}^{i}$ and $A_{t_{2}}^{i}$ are the urban areas of city *i* at times *t*_1_ and *t*_2_, respectively; $P_{t_{1}}^{i}$ and $P_{t_{2}}^{i}$ are the urban populations of city *i* at times *t*_1_ and *t*_2_. When the annual growth speed of the area of city *i* exceeds the annual growth speed of the urban population, USI > 0, while otherwise, USI < 0. USI ≤ 0 indicates that the city has not sprawled, and USI >0 indicates that it has sprawled.

### 3.2. Spatial Autocorrelation Model

This paper adopts the intensity of urban expansion as the initial observation value for the calculation of cold and hot spots [45]. The urban expansion intensity is employed as the initial observation value for the cold and hot spot calculations [45]. This paper introduces the Getis–Ord $G_{i}^{*}$ indices to measure the local clustering characteristics, respectively, of the urban sprawl in the UYR. The calculation formula is as follows:

$$G_{i}^{*}=\sum_{j=1}^{n}W_{ij}(d)X_{i}X_{j}/\sum_{j=1}^{n}X_{j}$$

where $G_{i}^{*}$ is the spatial weight defined by the distance rule, and the spatial weight of an adjacent research unit is 1, while that of a nonadjacent research unit is 0. *X_i_* and *X_j_* are the urban expansion intensities of area *i* and area *j*, respectively.

### 3.3. Urban Sprawl Scenario Analysis Model

This paper introduces the urban sprawl scenario analysis (USSA) model to simulate the urban sprawl conditions under different scenarios [27]. This model comprehensively considers the urban sprawl source, ecological barrier, ecological resistance, and urban sprawl extent. The calculation equation is as follows:

$$TV_{\mathrm{USSA}}=g_{\min}(\sum_{x=i}^{y=j}D_{xy}\times U_{x}\times R_{y}\times E_{y}),\;\mathrm{for}\;U_{x}=\left\{ \begin{array}{l} 0.8,\;\;\;\;\mathrm{USI}\geq V_{a}\\ 0.9,\;0\leq \mathrm{USI}<V_{a}\\ 1,\;\;\;\;\;\;\;\mathrm{USI}<0 \end{array}\right.$$

where ${\mathrm{TV}}_{\mathrm{USSA}}$ is the trend value of the USSA model. Negative function *g* is unknown, min is the cumulative minimum value of the different levels in each cell, $D_{\mathrm{xy}}$ is the length of the source *y* to the spatial grid of source *x*, $U_{x}$ is the USI in which *x* is situated, and $R_{y}$ is the relative damping coefficient of the rank of *y*. The greater the rank of *y* is, the stronger the capability of urban sprawl, and the lower the relative damping coefficient. $E_{y}$ is the ecological control factor of *y*. $V_{a}$ is the average value of the USI.

This paper identifies the sources of urban sprawl based on urban sprawl cold–hot spots from 2009 to 2015. The source is the root cause of urban land maintenance and sprawl and is the starting point and foundation of urban sprawl. Source refers to the municipal district formed by the development of a city over a certain period of time. We defined weights of 0.6, 0.7, 0.8, and 0.9, corresponding to the weight of the source in the hot spots, sub-hot, sub-cold, and cold spots, respectively, as shown in Figure S1. Sources are categorized to characterize differences in spatial sprawl capacity across municipal districts. Therefore, the comparative analysis in this paper is all about the extent of sprawl in urban municipalities, and rural areas are not included.

### 3.4. Urban Sprawl Influence Factor Model

#### 3.4.1. Geodetector

This paper cites Geodetector to analyze the factors influencing urban sprawl in the UYR. The Geodetector is a model developed by Wang et al. [46] to detect spatial heterogeneity and reveal the driving factors behind it. Factor detectors are used to identify the importance of the drivers affecting the spatial and temporal patterns of urban sprawl, and interaction detectors are used to explore the impact of interactions between drivers on urban sprawl. The core idea of using a Geodetector to study the influencing factors of urban sprawl is that if an influencing factor has a significant effect on the developmental changes of urban sprawl, then the spatial distribution of this factor and the degree of urban sprawl should have similarity [47]. The formula for the factor detector is as follows [48]:

$$q\;=\;1\;-\;\frac{\sum_{L}^{h=i}N_{h}\sigma_{h}^{2}}{N\sigma^{2}}$$

where *q* is the influence intensity value of the driver on urban sprawl and takes the value range [0, 1]. A higher *q*-statistic indicates a more significant effect of the factor on urban sprawl and vice versa. *L* is the number of classifications of influencing factors. *N_h_* is the number of cities in category *h*. *N* is the number of cities in the entire study area. *σ*_h_^2^ is the variance of the *h* class. *σ*^2^ is the variance of the full urban sprawl index.

The interaction detector first calculates the *q*-statistic of the two factors X1 and X2 for urban sprawl, namely *q*(X1) and *q*(X2), respectively, and calculates the *q*-statistic when they interact, namely *q*(X1 ∩ X2), and finally compares the calculated results. The types of interactions are shown in Table 1.

#### 3.4.2. Urban Sprawl Influence Indicator System Construction

The extent of urban sprawl and its spatial heterogeneity are influenced by a combination of many factors such as economic growth, social development, natural conditions, and policy making. Based on the principles of scientific, comparability, independence, operability, and accessibility of indicators and on the basis of existing results [49,50,51], 15 indicators were selected from four levels, namely economic development, social culture, transportation, and government regulation, to quantitatively analyze the drivers of urban sprawl in the UYR, as shown in Table 2. Using the natural break method in ArcGIS 10.8, the 15 metrics and USI were reclassified into 10 categories and sampled at 10 km × 10 km intervals and imported into the Geodetector.

## 4. Results

### 4.1. Recognition of the Spatial Features of Urban Sprawl

The USI was adopted in the four periods of 1992–1996, 1996–2002, 2002–2009, and 2009–2015. To facilitate comparative analysis, the USI from 2009 to 2015 was classified by a combination of artificial and natural fracture methods, and the classification criteria were applied to the first three periods.

**Figure 2:**
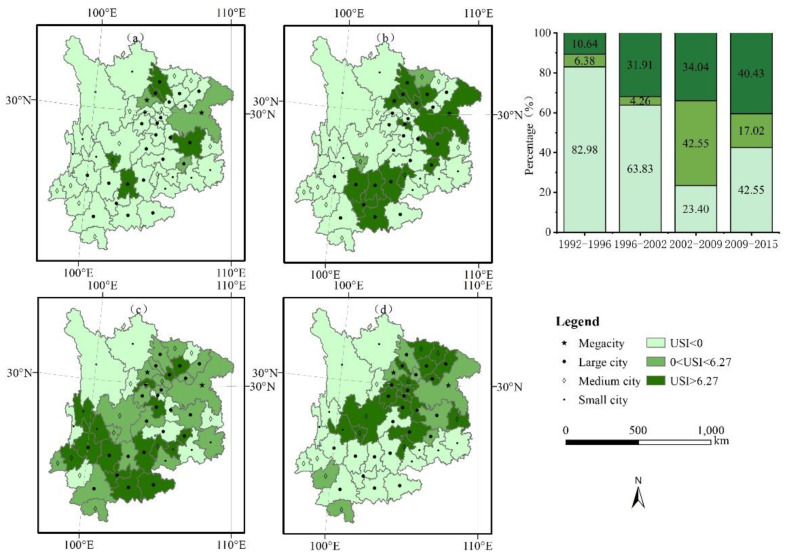
Urban sprawl across different cities in UYR, China, from 1992 to 2015: (**a**–**d**) the four periods of 1992–1996, 1996–2002, 2002–2009, and 2009–2015, respectively.

The USI in the UYR first increased and then stabilized over time (Figure 2). From 1992 to 1996, cities such as Deyang, Kunming, Panzhihua, Zunyi, and Mianyang experienced severe urban sprawl, accounting for 10.64% of the UYR. During this period, the cities without sprawl accounted for 82.98% of the UYR. From 1996 to 2002, Chengdu, Chongqing, Guiyang, and Nanchong developed into cities experiencing severe urban sprawl, accounting for 31.91% of the UYR, an increase of 21.28% over the previous period. The area without sprawl accounted for 63.83%, a decrease of 19.15% over the previous period. From 2002 to 2009, Chengdu, Chongqing, Kunming, Deyang, Mianyang, and Zunyi were converted into areas with a slight urban sprawl, while Meishan, Yibin, Dali, Lijiang, Anshun, etc., were newly added areas faced with severe urban sprawl. During this period, the area without urban sprawl in the UYR decreased to 23.40% of the entire study area. From 2009 to 2015, Leshan, Luzhou, and Guangan were newly identified as areas with severe urban sprawl. More than 40.43% of the UYR experienced severe urban sprawl, while the area with a slight urban sprawl decreased to 17.02% of the entire UYR. The area without urban sprawl added to 42.55% of the entire UYR.

### 4.2. The Pattern and Evolution of the Urban Sprawl Cold and Hot Spots

**Figure 3:**
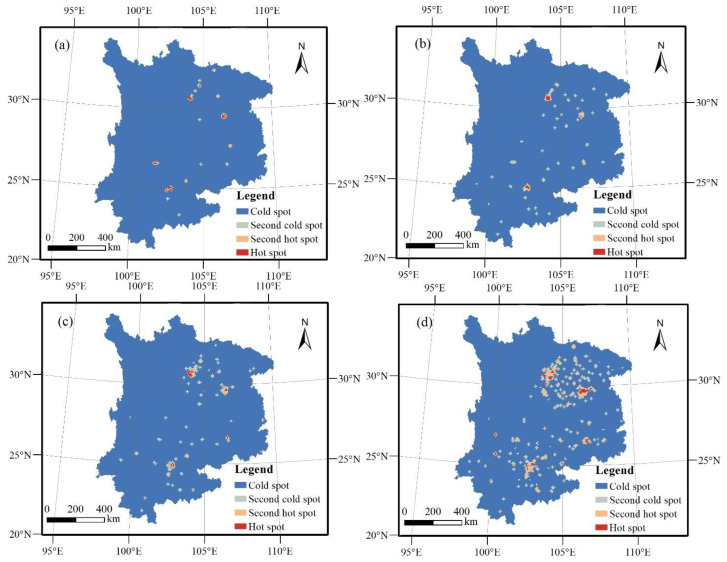
Distribution of urban sprawl cold and hot spots in the UYR, China, from 1992 to 2015: (**a**–**d**) the four periods of 1992–1996, 1996–2002, 2002–2009, and 2009–2015, respectively.

The Getis–Ord $G_{i}^{*}$ index is employed to analyze the local features of the cold and hot spots during the above four periods of the urban sprawl in the UYR. The Getis–Ord $G_{i}^{*}$ index value of the research unit was divided into four categories according to the natural fracture method, and they were denoted as hot spots, sub-hot spots, sub-cold spots, and cold spots in descending order, as shown in Figure 3.

From 1992 to 1996, the cold and hot spots of the urban sprawl in the UYR were clearly distinguished. The hot spots were mainly centered on Chengdu, Chongqing, Kunming, Mianyang, and Panzhihua, as illustrated in Figure 3a. From 1996 to 2002, the urban sprawl cold and hot spots in the UYR underwent major changes. The hot spots were mostly centered on Chengdu, Chongqing, and Kunming. Yuxi developed from a sub-cold spot into a sub-hot spot, and the area of the sub-cold spot continued to increase, as shown in Figure 3b. From 2002 to 2009, the distribution of the sub-hot spots increased. The newly added sub-hot spots included Nanchong, Suining, and Dali, as shown in Figure 3c. From 2009 to 2015, the layout of the hot spots of the urban sprawl in the UYR during this period was fragmented. New hot spots included Deyang, Meishan, Lijiang, Dali, and Southwest Guizhou, as shown in Figure 3d. The newly added hot-spot cities connected the sub-hot spot cities in the surrounding area, thereby forming a small radiation center and driving the development of the surrounding area.

### 4.3. The Simulation Results of the Different Urban Sprawl Scenarios

#### 4.3.1. Test of the Urban Sprawl Scenario Analysis Model

**Figure 4:**
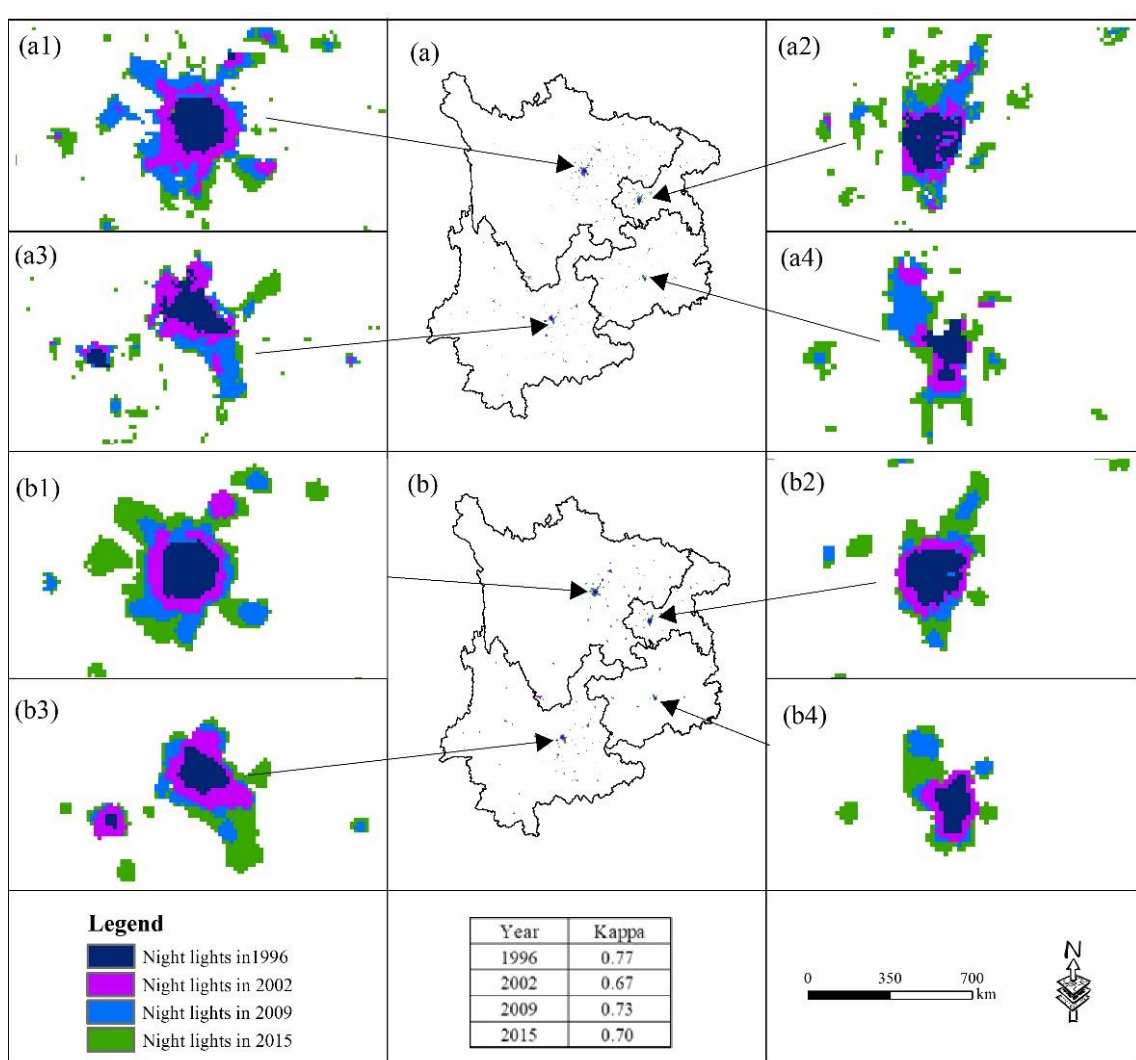
Calibrating the USSA model with the urban sprawl simulation in the UYR, China, from 1992 to 2015: (**a1**–**a4**) represent the near-real night light data of Chengdu, Chongqing, Kunming, and Guiyang, respectively; (**b1**–**b4**) represent the simulated night light data of Chengdu, Chongqing, Kunming, and Guiyang, respectively.

According to the established model, the nighttime data in 1992, 1996, 2002, and 2009 were adopted as sources to simulate the nighttime light distributions in 1996, 2002, 2009, and 2015. The kappa coefficient of the near-real night light data and simulated night light data was calculated, as shown in Figure 4.

Figure 4 shows a detailed comparison of the near-real nighttime light data (a) and the simulated nighttime light data (b) of the four phases. In addition, the kappa coefficient of the corresponding year is also calculated. The results indicate that the kappa coefficients of the nighttime light simulation results in 1996, 2009, 2002, and 2015 are all approximately 0.7, which is highly consistent with the real results. Therefore, the model constructed in this study basically satisfies the research needs.

#### 4.3.2. Scenario Simulation of the Different Scales of Urban Sprawl

**Figure 5:**
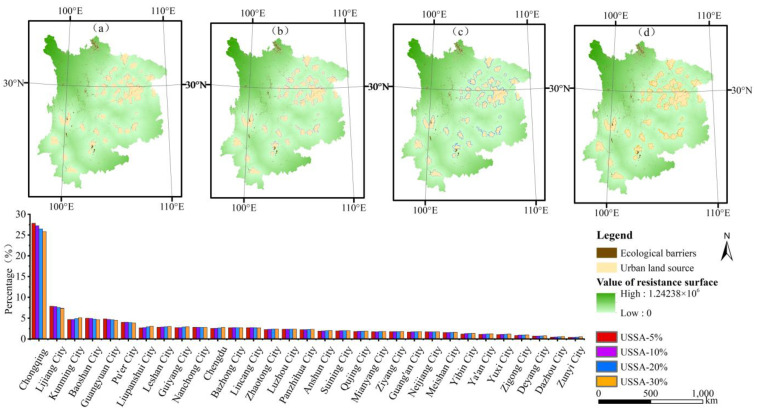
Simulation results of urban sprawl under different scenarios in the UYR, China: (**a**–**d**) represent the boundary when the simulated urban sprawl to 5%, 10%, 20%, and 30%, respectively.

The constructed ecological resistance surface was employed to simulate urban sprawl. The specific method involved the use of the grid reclassification function in ArcGIS with the percentage of the urban sprawl scale in the total study area in the classifier to obtain the boundaries of urban sprawl. It is known that the total area of the municipal district in the UYR amounts to 92,392.35 km^2^. This paper set four different scenarios to simulate the urban sprawl boundaries. Scenario one, scenario two, scenario three and scenario four below represent the simulated urban sprawl to 5%, 10%, 20%, and 30%, respectively. Moreover, the urban space area accounts for 21.88%, 29.22%, 51.60%, and 72.50%, respectively, of the total study area, as shown in Figure 5.

In this study, the urban sprawl pattern refers to a spatial characteristic and spatial structure formed by the urban sprawl process. Under scenario one and scenario two, the urban boundary has sprawled, exhibiting a scattered layout, as shown in Figure 5a and Figure 5b, respectively. Largely owing to the restriction of ecological barriers, the urban boundary occurs away based on the urban centers. Under scenario three, the urban sprawl increases along the original trend, and the urban spatial form changes into the marginal type, while the municipal district becomes surrounded by newly sprawled areas, as shown in Figure 5c. Under scenario four, the urban spatial form reveals a concentric belt structure, and the municipal district has become completely surrounded by newly sprawled areas, as shown in Figure 5d. It can be seen that the urban sprawl pattern under different scale scenarios shows a transformation of “ decentralized layout-edge type-concentric belt”.

To more clearly reflect the sprawl rate of each city at different scales, statistics were obtained for the various cities. The cities with the highest degree of sprawl are Chongqing, Lijiang, and Kunming, which account for 25.84%, 7.37%, and 5.11%, respectively, of the UYR, as shown in Figure 5.

#### 4.3.3. Scenario Simulation of the Urban Sprawl at Different Times

**Figure 6:**
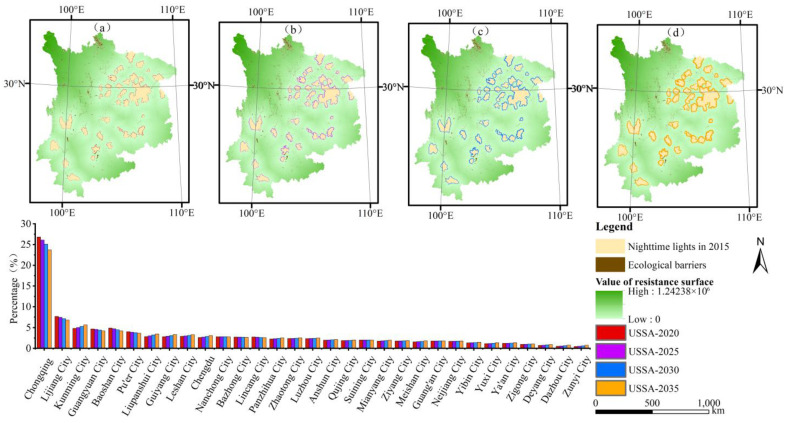
Simulation results of urban sprawl under different years in UYR, China: (**a**–**d**) the boundaries of the simulated urban sprawl to 2020, 2025, 2030, and 2035, respectively.

Based on the obtained continuous nighttime light remote sensing data, this paper applied the grey prediction method to conduct urban sprawl simulations in 2020, 2025, 2030, and 2035. The results showed that the urban land area in these four years was 13,750 km^2^, 23,580 km^2^, 40,460 km^2^, and 69,400 km^2^, respectively, at an annual growth rate of 5.45%, as shown in Figure 6.

Figure 6 shows that similar sprawl trends occurred in different time simulations. Over time, the urban spatial form also underwent a transformation of “decentralized layout-edge type-concentric belt”. Overall, the degree of sprawl in the different time scenario simulations is higher than that in the varying scale scenario simulations. From the perspective of a single city, the degree of sprawl in the different time scenario simulations in cities such as Chongqing, Lijiang, and Guangyuan is lower than that in the different scale scenario simulations, while the degree of sprawl in cities such as Kunming, Guiyang, and Liupanshui in the different time scenario simulations is higher than that in the different scale scenarios. The cities with the highest degree of sprawl are Chongqing, Lijiang, and Kunming, which account for 23.72%, 6.80%, and 5.65%, respectively, of the UYR, as shown in Figure 6.

### 4.4. Analysis of Urban Sprawl Influencing Factors

#### 4.4.1. Analysis of Urban Sprawl Influence Factor Detectors

**Figure 7:**
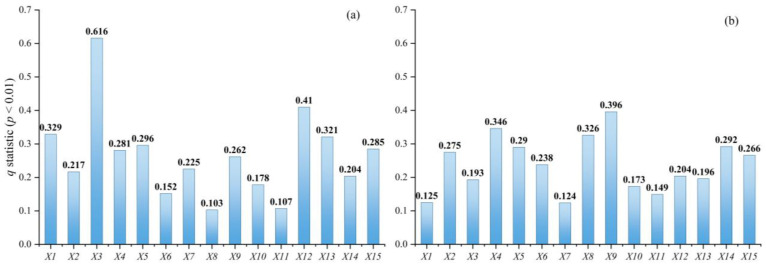
The *q*-statistic of each driving factor in different years: (**a**) 1996–2002; (**b**) 2009–2015.

The factor detector measures the effect of each factor on the extent of urban sprawl. As shown in Figure 7, from 1996 to 2002, the *q*-statistic of the impact factors ranged from 0.103 to 0.616 and were all significant (*p* < 0.01). The effects on urban sprawl are, from strong to weak, as follows: secondary industry share of GDP > private car ownership > GDP > highway mileage > investment in real estate development > fixed asset investment > tertiary industry share of GDP > urban green space per capita > urbanization rate > urban disposable income per capita > public finance expenditure > urban road area per capita > population > distance from major railroads > number of high schools. From 2009 to 2015, the *q*-statistic of the impact factors ranged from 0.124–0.396 and were all significant (*p* < 0.01). The effects on urban sprawl from strong to weak are: urban green space per capita > tertiary industry share of GDP > number of high schools > public finance expenditure > investment in real estate development > urban disposable income per capita > fixed asset investment > population > private car ownership > highway mileage > secondary industry share of GDP > urban road area per capita > distance from major railroads > GDP > urbanization rate.

From a factor perspective, from 1996 to 2002, the driver with the highest influence on urban sprawl was secondary industry share of GDP, with a *q*-statistic of 0.616. The higher the level of secondary industry development in a city, the higher the extent of urban sprawl that follows. The effect of private car ownership on urban sprawl is the second with a *q*-statistic of 0.41. Private car ownership can indicate the level of urban accessibility, and as cities become more accessible, residents are more inclined to live in the suburbs, thus indirectly growing urban sprawl. The convenience of transportation will also reduce the cost of transportation and land rent for developers, resulting in overdevelopment of the peripheral areas of the city. From 2009 to 2015, the driver with the highest influence on urban sprawl was green space per capita with a *q*-statistic of 0.396. The higher the green space per capita, the more residents tend to live in places with a high quality of urban life, thus contributing to urban sprawl. This finding is consistent with that of Koprowska et al. [26], who also concluded that urban green spaces contribute to urban sprawl. We can also observe the higher influence of the tertiary industry share of GDP than the secondary industry share of GDP during this period. With the development of the tertiary industry, residents are attracted to live in areas with a strong service sector, thus contributing to urban sprawl.

From a dimensional perspective, from 1996 to 2002, the influence of economic development on urban sprawl was clearly dominant. However, by 2009 to 2015, there is no significant difference in the *q*-statistic of the dimensions, indicating that the dimensions have comparable influence on urban sprawl, and there is no particularly significant dominant influence factor.

#### 4.4.2. Analysis of Urban Sprawl Influence Factor Interaction Detectors

**Figure 8:**
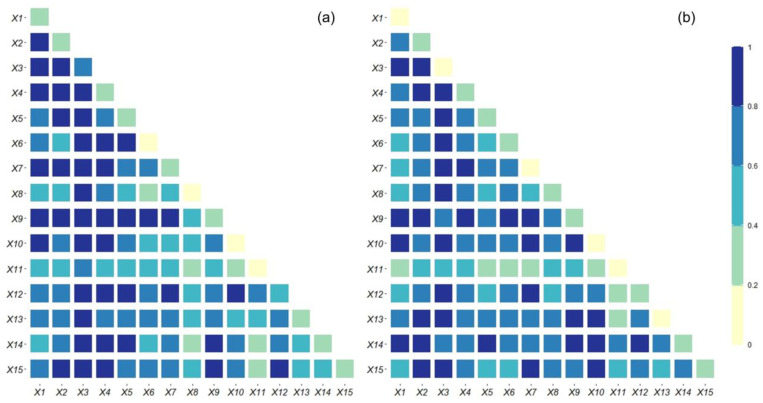
Interactive detection of driving factors. (**a**) 1996–2002; (**b**) 2009–2015.

Urban sprawl is a geographical process in which multiple factors interact. Interaction detectors can measure the effect of interactions of various factors on urban sprawl. As shown in Figure 8, after the interaction of the influencing factors, the *q*-statistic increases significantly, and the impact on urban sprawl is also significantly enhanced. The interaction detector judgment condition showed that from 1996 to 2002, the number of two-factor enhancements was 12, and the number of non-linear enhancements was 93. From 2009 to 2015, the number of two-factor enhancements was 9, and the number of non-linear enhancements was 96. This indicates that the interaction effect of any two influencing factors on urban sprawl is an enhancing relationship, and there are no mutually independent influencing factors, and the influence of each factor on urban sprawl is somewhat correlated, and all of them have a higher degree of influence than individual factors.

From 1996 to 2002, the interaction between urban disposable income per capita and urbanization rate has the highest effect on urban sprawl with a *q*-statistic of 0.98. The interaction of the number of high schools and distance from major railroads has the lowest effect on urban sprawl with a *q*-statistic of 0.26. The interaction effect of the secondary industry share of GDP on the other factors is significantly enhanced with *q*-statistic ranging from 0.72–0.97, indicating that the secondary industry share of GDP significantly enhances the effect of the other factors on urban sprawl. From 2009 to 2015, the interaction between the secondary industry share of GDP and the population has the highest effect on urban sprawl with a *q*-statistic of 0.97. The interaction between GDP and distance from major railroads has the lowest effect on urban sprawl with a *q*-statistic of 0.33. The interaction effect of urban disposable income per capita on other factors is significantly stronger with *q*-statistic ranging from 0.48–0.9, indicating that disposable income per capita amplifies the effect of other factors on urban sprawl. Comparing the two time periods, the interaction power of the number of high schools on other factors increases from 0.26–0.55 to 0.47–0.8, indicating that the improvement of education quality can have a stronger influence in combination with other factors.

## 5. Discussion

### 5.1. The Scale Effect of Urban Sprawl Spatial Pattern Evolution

**Figure 9:**
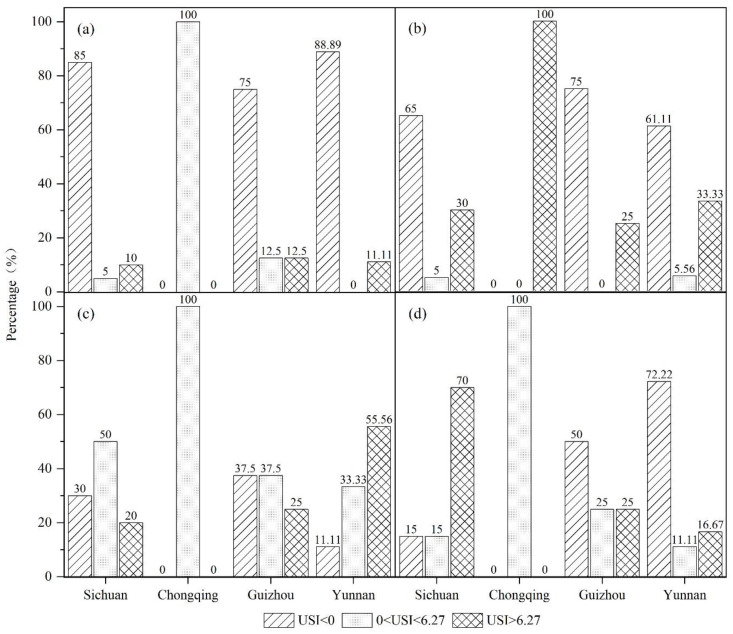
Urban sprawl across different provinces in UYR, China, from 1992 to 2015: (**a**–**d**) the four periods of 1992–1996, 1996–2002, 2002–2009, and 2009–2015, respectively.

**Figure 10:**
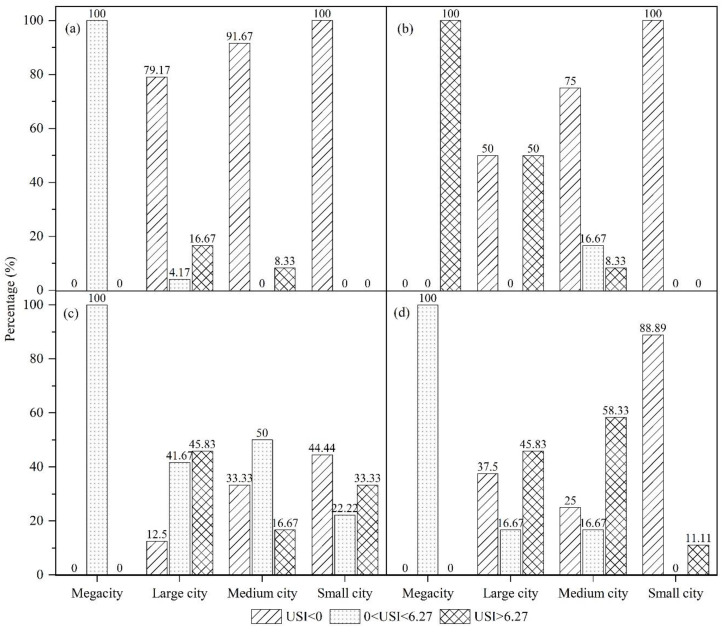
Urban sprawl across different city scales in UYR, China, from 1992 to 2015: (**a**–**d**) the four periods of 1992–1996, 1996–2002, 2002–2009, and 2009–2015, respectively.

**Figure 11:**
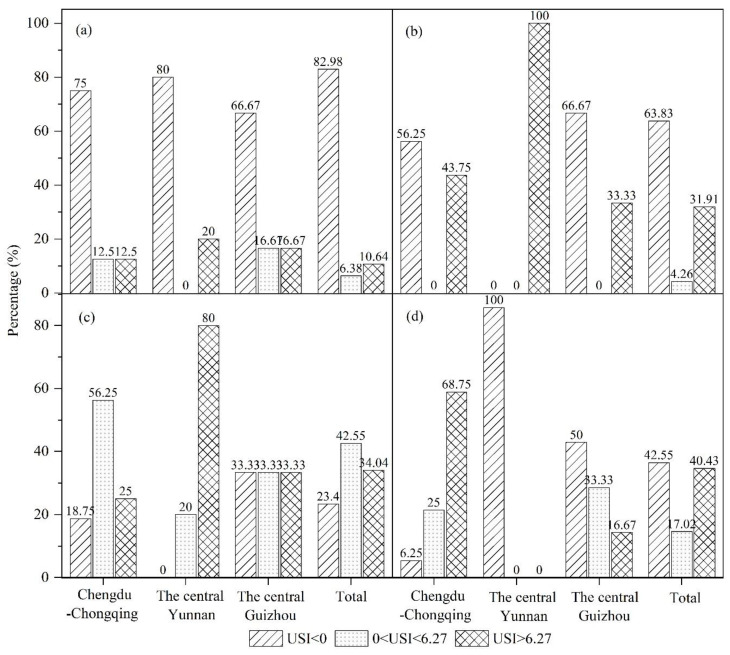
Urban sprawl across different urban agglomerations in UYR, China, from 1992 to 2015: (**a**–**d**) the four periods of 1992–1996, 1996–2002, 2002–2009, and 2009–2015, respectively.

The USI was determined during the four time periods of 1992–1996, 1996–2002, 2002–2009, and 2009–2015 to conduct a multiscale discussion of the characteristics of the urban sprawl in the UYR (Figure 9, Figure 10 and Figure 11). The following three spatial scales are used in this study: city scale (megacity, large city, medium city, small city), urban agglomeration scale (Chengdu-Chongqing urban agglomeration, the central Yunnan urban agglomeration, the central Guizhou urban agglomeration), and provincial administrative scale (Chongqing, Sichuan, Yunnan, Guizhou).

From 1992 to 1996, with the exception of Chongqing, the other three provinces experienced severe urban sprawl. Among them, the areas experiencing severe sprawl in Sichuan Province accounted for 10% of the province, the areas with slight sprawl accounted for 5% of the province, and the remaining 85% of the areas did not experience urban sprawl. In Guizhou Province, the areas suffering severe sprawl accounted for 12.5% of the total province, the areas with slight sprawl accounted for 12.5% of the total province, and the remaining 75% of the areas did not reveal urban sprawl. The areas with severe sprawl in Yunnan Province accounted for 11.1% of the whole province, and the remaining 88.89% of the areas did not experience sprawl, as shown in Figure 9a. From 1996 to 2002, Chongqing developed into a severely sprawling city. The areas experiencing severe sprawl in Sichuan accounted for 30% of the province, an increase of 20% over the previous period. The areas exhibiting severe sprawl in Guizhou Province accounted for 25% of the province, an increase of 12.5% over the previous period. The areas with severe sprawl in Yunnan Province accounted for 33.33% of the province, an increase of 8.33% over the previous period, as shown in Figure 9b. From 2002 to 2009, Chongqing experienced slight sprawl. The proportion of slight sprawl in Sichuan Province rose to 50%, an increase of 45% over the previous period, while the proportion of severe sprawl fell to 20%, a decrease of 10% over the previous period. The proportion of slight sprawl in Guizhou and Yunnan provinces rose to 37.5% and 33.33%, respectively, as shown in Figure 9c. From 2009 to 2015, Chongqing continued to experience slight sprawl. The area with severe sprawl in Sichuan Province accounted for 70% of the province, an increase of 50% over the previous period, and the proportion of sprawl increased to 85%, reaching a sprawl peak. The phenomenon of urban sprawl in Sichuan Province has continuously exhibited an upward trend. The proportion of sprawl in Guizhou Province and Yunnan Province decreased to 50% and 27.78%, respectively, and they began to show a downward trend, as shown in Figure 9d.

From 1992 to 1996, the phenomenon of urban sprawl in megacities became the dominant phenomenon. The proportion of sprawl in the large cities was 20.84%, of which 16.67% were areas with severe sprawl, and 4.17% were areas experiencing slight sprawl. The proportion of the regions suffering severe sprawl in the medium cities was 8.33%, while urban sprawl did not occur in small cities, as seen in Figure 10a. From 1996 to 2002, the urban sprawl in megacities increased. Among the large cities, the proportion of severe sprawl reached 50%, an increase of 33.33% over the previous period. The proportion of sprawl in the medium cities was 25%, an increase of 16.67% over the previous period, of which 8.33% were severely sprawled, and 16.67% were slightly sprawled. No urban sprawl occurred in the small cities, as shown in Figure 10b. From 2002 to 2009, the USI in megacities was mainly moderate. The urban sprawl during this period mainly occurred in large cities. The proportion of USI in the large cities was 87.5%, an increase of 37.5% over the previous period. Among them, the number of areas exhibiting severe sprawl accounted for 45.83% of the total area, and the areas with slight sprawl accounted for 41.67% of the total area. The proportion of the medium cities with sprawl reached 66.67%, an increase of 41.67% over the previous period. Among them, the number of areas suffering severe sprawl accounted for 16.67% of the total area, and the number of areas with slight sprawl accounted for 50% of the total area. In the small cities, the area with severe sprawl accounted for 33.33% of the total area, and the area experiencing slight sprawl accounted for 22.22% of the total area, as shown in Figure 10c. From 2009 to 2015, the sprawl in megacities was predominantly slight, and the degree of sprawl in the large cities began to decline. The medium cities contained the largest number of cities with sprawl. The areas exhibiting severe sprawl accounted for 58.33% of the total area, and the areas with slight sprawl accounted for 16.67% of the total area. The degree of sprawl in the small cities began to decrease, accounting for 11.11% of the overall sprawl, a decrease of 44.44% over the previous period, as shown in Figure 10d.

From 1992 to 1996, the number of cities in the Chengdu-Chongqing urban agglomeration area accounted for 25% of the entire Chengdu-Chongqing area, of which the lightly sprawling areas accounted for 12.5%, and the severely sprawling areas accounted for 12.5% of the total area. The urban agglomeration in central Yunnan experienced severe sprawl occupied for 20% of the central Yunnan. The urban agglomeration of central Guizhou suffered severe sprawl accounted for 16.67% of the entire central Guizhou area, as shown in Figure 11a. From 1996 to 2002, the area of the Chengdu-Chongqing urban agglomeration exhibited severe sprawl accounted for 43.75% of the entire Chengdu-Chongqing area, an increase of 31.25% over the previous period. The overall evolution of the central Yunnan urban agglomeration involved severe sprawl. The urban agglomeration in the central Guizhou developed into a severely sprawled area accounted for 33.33% of the entire central Guizhou area, an increase of 16.67% over the previous period. During this period, the three major urban agglomerations presented a relatively fast urban sprawl speed, as shown in Figure 11b. From 2002 to 2009, the area where Chengdu-Chongqing urban agglomeration sprawl occurred accounted for 81.25% of the entire Chengdu-Chongqing area, an increase of 37.50% over the previous period. The urban agglomeration in central Yunnan experienced sprawl as a whole, of which 80% was severe sprawl, and 20% was slight sprawl. The urban agglomeration in central Guizhou accounted for the same proportions of severe sprawl, slight sprawl, and no sprawl, as shown in Figure 11c. From 2009 to 2015, the degree of sprawl in the Chengdu-Chongqing urban agglomeration continued to increase, and the sprawled region is occupied for 93.75% of the Chengdu-Chongqing urban agglomeration, as shown in Figure 11d. There was no urban sprawl in the central Yunnan urban agglomeration. The sprawl in the urban agglomeration area in central Guizhou also decreased, and the amount of urban sprawl decreased by 16.67% over the previous period.

### 5.2. The Scale Effect of the Urban Sprawl under Different Time Scenarios

Similarly, to more clearly reflect the differences in urban sprawl between the different scales, statistical analysis was conducted at the various city scales and different urban agglomeration scales. At the different urban scales, the large cities exhibited the largest sprawl, with a net increase of 26,808.61 km^2^ from 6580.47 km^2^ in 2020 to 33,389.07 km^2^ in 2035, accounting for approximately 48.16% of the sprawl of the entire study area, as indicated in Table 3. From 2020 to 2035, the small cities revealed the lowest sprawl, with a net growth of 4199 km^2^, accounting for approximately 7.54% of the sprawl of the entire study area. Among them, Chongqing exhibited the highest urban sprawl from 2020 to 2035, increasing from 2415.742 km^2^ to 12,332.95 km^2^, accounting for 23.72% of the UYR. Zunyi city exhibited the lowest urban sprawl, adding from 157.28 km^2^ in 2020 to 919.28 km^2^ in 2035, accounting for 1% of the UYR.

At the different scales of the urban agglomerations, the three major urban agglomerations all experienced severe urban sprawl, as indicated in Table 3. From 2020 to 2035, the urban sprawl area in the Chengdu-Chongqing urban agglomeration increased from 6987.412 km^2^ to 35,299.412 km^2^, a net increase of 28,312 km^2^, accounting for 50.92% of the sprawl of the entire study area. From 2020 to 2035, the urban sprawl area in the central Yunnan urban agglomeration expanded from 1550.725 km^2^ to 7858.725 km^2^, a net increase of 6308 km^2^, accounting for 11.34% of the sprawl of the entire study area. From 2020 to 2035, the urban sprawl area in central Guizhou added from 1165.393 km^2^ to 5468.393 km^2^, a net increase of 4483 km^2^, accounting for 8.15% of the sprawl of the entire UYR. It can be summarized that the Chengdu-Chongqing urban agglomeration experienced the largest sprawl trend, while the Guizhou urban agglomeration exhibited the smallest sprawl trend.

## 6. Conclusions

This study measured the level and characteristics of urban sprawl under different scales; assessed the spatial pattern change law of urban sprawl from 1992 to 2015; simulated the evolution trend of different scenarios in 2020, 2025, 2030, and 2035; and analyzed the influence of driving factors and factor interactions on urban sprawl in different periods. The proportions of the areas where urban sprawl occurred during the four time periods of 1992–1996, 1996–2002, 2002–2009, and 2009–2015 were 17.02%, 36.17%, 76.59%, and 57.45%, respectively. Thus, urban sprawl conditions were observed in the UYR from 1992 to 2015, but urban sprawl fluctuated significantly over time, showing an intensification from 1992 to 2009 and a weakening from 2009 to 2015.

From 1992 to 2009, the hot spots of urban sprawl in the UYR were mainly concentrated in Chengdu, Chongqing, Kunming, and Guiyang, showing signs of sprawl dominated by individual cities. From 2009 to 2015, the cities around these four cities were added as new hot spots. The newly added hot-spot cities connected the sub-hot-spot cities in the surrounding area, thereby forming a small radiation center and driving the development of the surrounding area. The layout of the hot spots of the urban sprawl was fragmented.

In the scenario simulations at the different scales, the spatial pattern of urban sprawl in the UYR from 1992 to 2015 underwent a transformation of “decentralized layout-edge type-concentric belt”. The cities with the highest degree of sprawl were Chongqing, Lijiang, and Kunming, with their sprawled areas accounting for 25.84%, 7.37%, and 5.11%, respectively, of the UYR. Under the different time scenarios, the large cities revealed the most sprawl, accounting for more than 47% of the UYR, and the small cities experienced the least sprawl, accounting for less than 7.6% of the UYR. The Chengdu-Chongqing urban agglomeration attained the largest sprawled area, accounting for 50.92% of the UYR.

The driving factor with the highest influence on urban sprawl changed from the secondary industry share of GDP from 1996 to 2002 to the green space per capita from 2009 to 2015. Residents prefer to live in areas with complete urban infrastructure and high quality of life, which contributes to urban sprawl. The interaction of any two influencing factors on urban sprawl is higher than the influence of a single factor. The number of high schools interacts significantly higher compared to the other factors, indicating that the improvement of education quality can have a stronger influence on urban sprawl in combination with other factors.

This study is based on the concept of “identification—spatial pattern—scenario simulation—influencing factors” to simulate the evolution of future urban sprawl and to analyze the influencing factors of urban sprawl and the interaction of factors in different time periods The study contributes to policy makers understanding of the state of urban development. It is conducive to planning a more rational urban spatial layout, targeting to curb disorderly urban sprawl, and achieving smart and rational growth of urban space and sustainable and competitive healthy urban development.

Urban sprawl to accommodate growing urban populations has caused cities to expand spatially beyond their boundaries into their hinterlands and surrounding areas, which means that urban sustainability and population health is reduced. Currently, China has adopted spatial planning measures to control uncontrolled urban sprawl and to protect the land and ecological environment. Although overall urban sprawl has slowed in recent years, it remains severe in major large cities. We suggest setting urban spatial growth boundaries in large cities and clarifying the boundaries of non-construction land such as agricultural protection land, water protection land, natural habitat, and development reserves in the region. We recommend spatial land planning as a whole to control land concessions, increase the compactness of land use, reasonably distribute the layout and density of production and living land, and improve the land-use efficiency.

## Figures and Tables

**Table 1:** Types of influence factor interaction.

Judgment Conditions	Interaction
*q*(X1∩X2) < Min(*q*(X1),*q*(X2))	Non-linear reduction
Min(*q*(X1),*q*(X2)) < *q*(X1∩X2) < Max(*q*(X1),*q*(X2))	Single-factor nonlinearity reduction
*q*(X1∩X2) > Max(*q*(X1),*q*(X2))	Two-factor enhancement
*q*(X1∩X2) = *q*(X1) + *q*(X2)	Independent
*q*(X1∩X2) > *q*(X1) + *q*(X2)	Non-linear enhancement

**Table 2:** Urban sprawl influence indicator system.

Dimensions	Factors	Code	Unit
Economic development	GDP	*X1*	Billion CNY
	Urban disposable income per capita	*X2*	CNY
	Secondary industry share of GDP	*X3*	%
	Tertiary industry share of GDP	*X4*	%
	Investment in real estate development	*X5*	Billion CNY
Social culture	Population	*X6*	Million
	Urbanization rate	*X7*	%
	Number of high schools	*X8*	-
	Urban green space per capita	*X9*	m^2^
Transportation	Urban road area per capita	*X10*	m^2^
	Distance from major railroads	*X11*	m
	Private car ownership	*X12*	-
	Highway mileage	*X13*	km
Government regulation	Public finance expenditure	*X14*	Billion CNY
	Fixed assets input	*X15*	Billion CNY

**Table 3:** The sprawl rate of the urban under the four scenarios.

Scale Type		2020	2025	2030	2035
Urban size	Megacity	3001.275	5204.275	8927.358	15,101.67
		21.88%	22.13%	22.09%	21.78%
	Large city	6580.468	11,244.46	19,326.38	33,389.08
		47.98%	47.81%	47.83%	48.16%
	Medium city	3101.561	5298.561	9081.561	15,606.56
		22.61%	22.53%	22.47%	22.51%
	Small city	1031.342	1772.342	3072.342	5230.342
		7.52%	7.54%	7.60%	7.54%
Urban agglomeration	Chengdu-Chongqing	6987.412	12,089.41	20,684.41	35,299.41
		50.95%	51.40%	51.19%	50.92%
	The central Yunnan	1550.725	2652.725	4549.725	7858.725
		11.31%	11.28%	11.26%	11.34%
	The central Guizhou	1165.393	1921.393	3275.393	5648.393
		8.50%	8.17%	8.11%	8.15%

## Data Availability

The data presented in this study are available on request from the corresponding author.

## Supplementary Materials

The following supporting information can be downloaded at: https://www.mdpi.com/article/10.3390/ijerph19159190/s1, Figure S1: Classification of urban municipal sources.
