# Supplementary figures and images for: Simulation on the Evolution Trend of the Urban Sprawl Spatial Pattern in the Upper Reaches of the Yangtze River, China
Source: Int J Environ Res Public Health. 2022 Jul 27;19(15):9190. doi: 10.3390/ijerph19159190 (PMC9368520; doi:10.3390/ijerph19159190)

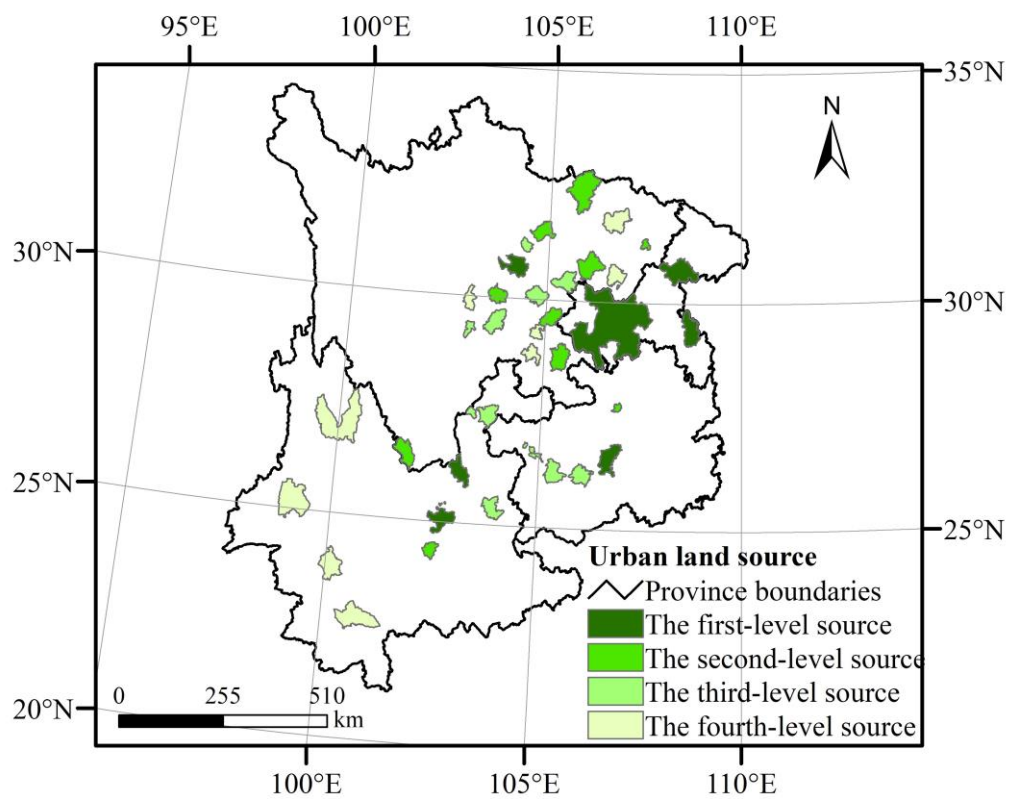

**Figure S1.** Classification of urban municipal sources.

Supplement: Supplementary file 1 [file ijerph-19-09190-s001.zip › ijerph-1718372-supplementary.pdf]
